# Datasets for assessing the structure and drivers of biological sounds

**DOI:** 10.1016/j.dib.2022.107930

**Published:** 2022-02-07

**Authors:** Johan Diepstraten, Jacques Keumo Kuenbou, Jacob Willie

**Affiliations:** a Animal Behaviour and Cognition, Departement of Biology, Faculty of Science, Utrecht University, the Netherlands; b Association de la protection des grands singes, Cameroon; c Centre for Research and Conservation, Royal Zoological Society of Antwerp, Belgium

**Keywords:** Soundscape, Soundscape drivers, Tropical rainforest, Passive acoustic monitoring, Recordings, Call Libraries

## Abstract

Obtaining and analysing sound data can be a tedious and lengthy process. We present sound data consisting of 20,485 1 min sound recordings obtained in three sites within a rainforest landscape in southeast Cameroon. The sites differ in anthropogenic disturbance. We also present meta data corresponding to these recordings with the identification of all animal vocalisations in each 1 min sound recording. Additionally, we provide a raw database with data on habitat, human activities, remoteness, accessibility, temperature, humidity, rainfall, moon phase, and mammal and bird observations in the area during the recording period. The data were used by Diepstraten & Willie (2021) to investigate the structure and drivers of biological sounds along a disturbance gradient. The data contribute to call libraries of tropical species and can also be used to build classifiers for automatic detection and classification of animal vocalisations.

## Specifications Table

**Table:** 

Subject	Ecology
Specific subject area	Soundscape ecology
Type of data	Table Audio
How data were acquired	Acoustic data were acquired through passive acoustic monitoring with the use of Audiomoth bioacoustics sensors. Local field assistants with an expertise in fauna of the study area detected and identified vocalisations in the recordings. Data on habitat, human activities, and mammal and bird occurrence were collected during transect surveys. Data on temperature, humidity, rainfall, and moon phase were collected in the area during the recording period. Accessibility and remoteness were calculated using coordinates in ARCGIS.
Data format	Raw Analysed
Parameters for data collection	Data were collected between February and May 2020 in the northern part of the Dja Faunal Reserve's buffer zone in Cameroon, at the start of the wet season. Data were obtained in three study sites that represent a gradient of disturbance. In each of the sites, six transects of 1 km each were opened for data collection. A sound recorder was placed in the middle of each transect.
Description of data collection	All sensors were set to record the first minute of every hour. All recordings were played to local experts who detected and identified all animal vocalisations. Furthermore, all transects were surveyed to obtain data on habitat, human activities, and animal occurence in each transect. Temperature and humidity were measured hourly in a fixed location of the study area. The amount of rainfall and moon phase were noted daily. Accessibility and remoteness were calculated for every sensor as the distance to the nearest trail and village, respectively.
Data source location	Institution: Centre for Research and Conservation, Royal Zoological Society of Antwerp City/Town/Region: Antwerp, Flanders Country: Belgium Latitude and longitude (and GPS coordinates, if possible) for collected samples/data: 290,790 371,088 (La Belgique); 284,481 384,796 (La Palestine); 266,412 390,111 (Ngouleminanga)
Data accessibility	Repository name: Dryad Direct URL to data: https://datadryad.org/stash/share/ewDRpKPWOQ26Zj3afnbeOuCP5QzIVwOW9sv_9wUGoCQ. Instructions for accessing these data: Data are uploaded to Dryad in “Private for Peer Review” mode. Reviewers can use the URL for a double-blind download of the dataset.
Related research article	Diepstraten, J., & Willie, J. (2021). Assessing the structure and drivers of biological sounds along a disturbance gradient. *Global Ecology and Conservation, 31*, e01819. https://doi.org/10.1016/j.gecco.2021.e01819 Diepstraten, J., Kuenbou, J. K., & Willie, J. (2022). Methods to measure biological sounds and assess their drivers in a tropical forest. *MethodsX*, 101,619. https://doi.org/10.1016/j.mex.2022.101619

## Value of the Data

• The files provide substantial amount of acoustic data with corresponding identifications of vocalising animals and ecological and anthropogenic factors from an understudied region.
• Ecologists and conservationists can benefit from these data that are otherwise difficult and time consuming to obtain.
• These data can be used for many purposes, including the analysis of the structure and drivers of a soundscape and the calculation of acoustic indices. The data can also be used to build classifiers for automatic detection and classification of animal vocalisations.

## 1 Data Description

FOLDERS

The folders contain audio files recorded from February to June 2020. There are 38,065 audio files recorded by 18 sensors, including 20,413 1 min audio files used in the study by Diepstraten & Willie [1,3]. The name of each folder corresponds to the name of the sensor that recorded the audio files.

FILES

‐ File: **Number of analysed audio files** (files included in the study by Diepstraten and Willie [1,3]).
  ○ **Transect:** Number of the transect where sensor was located.
  ○ **BT:** Number of files for each sensor in study site La Belgique.
  ○ **PT:** Number of files for each sensor in study site La Palestine.
  ○ **NT:** Number of files for each sensor in study site Ngouleminanga.
  ○ **Total:** Total number of audio files for all study sites together.
‐ File: **Local English and scientific names of animal species**
  ○ **Badjué:** Name of species in local language.
  ○ **English:** English name of species.
  ○ **Scientific:** Latin name of species.
  ○ **Book The birds of Cameroon (p.):** Page of species (if bird) in Languy, M. (2019). The Birds of Cameroon. Their status and distribution. Series ‘Studies in Afrotropical Zoology’, vol.299. Tervuren: Royal Museum for Central Africa.
  ○ **Book Birds of Western Africa (p.):** Page of species (if bird) in Borrow, N., & Demey, R. (2002). Birds of Western Africa. Series ‘Helm Identification Guides’. Gardners Books.
‐ File: **Identification of animal species in audio files:** Each row describes a vocalisation as detected in the sound recordings. These recordings are those included in the study by Diepstraten & Willie [1,3]**.** Thus, recordings with multiple vocalisations are split into multiple rows. The site, sensor, and file name can be used to locate the corresponding audio file in the repository.
  ○ **Site**: Site where recording was made.
  ○ **Sensor:** Code of sensor that recorded audio.
  ○ **Time of Recording:** Date and time of recording.
  ○ **File name**: Name of the audio file in the folder of the corresponding sensor.
  ○ **Identified_species_Assistant_1:** Identification of the species by the first local expert.
  ○ **Identified_species_Assistant_2:** Identification of the species by the second local expert.
  ○ **Consensus:** If the local experts did not agree on identification, the consensus is written here.
  ○ **Unspecified:** Identifications that could not be made to species level.
  ○ **Comments:** Remarks about the audio.
  ○ **Additional comments:** Extra comments about the audio.
  ○ **Conclusion:** Final decision regarding the vocalisation described in that row.
‐ File: **Vegetation types**
  ○ **Data Collector:** Person(s) in charge of data collection.
  ○ **Assistant:** Person(s) assisting with data collection.
  ○ **Study_site:** Site of data collection.
  ○ **Transect_number:** number of the transect where data were collected.
  ○ **Transect:** Code of the transect where data were collected.
  ○ **Date:** Date of data collection.
  ○ **Location_along_transect (m):** Location along the 1 km transect where data were collected.
  ○ **Vegetation_type:** Code of vegetation type observed.
  ○ **Comments:** Comments about the observation.
‐ File: **English names of vegetation types**
  ○ **English_name:** English name corresponding to the codes of the observed vegetation types.
  ○ **Vegetation_type_code:** Code of vegetation type.
‐ File: **Indirect observations of human activity**
  ○ **Data Collector:** Person(s) in charge of data collection.
  ○ **Assistant:** Person(s) assisting with data collection.
  ○ **Study_site:** Site of data collection.
  ○ **Transect_number:** number of the transect where data were collected.
  ○ **Transect:** Code of the transect where data were collected.
  ○ **Date:** Date of data collection.
  ○ **Location_along_transect (m):** Location along the 1 km transect where data were collected.
  ○ **Type_of_human_sign:** Type of human sign observed.
  ○ **Vegetation_type:** Code of vegetation type observed around the human sign.
‐ File: **Indirect observations of mammal signs**
  ○ **Data Collector:** Person(s) in charge of data collection.
  ○ **Assistant:** Person(s) assisting with data collection.
  ○ **Study_site:** Site of data collection.
  ○ **Transect_number:** Number of the transect where data were collected.
  ○ **Transect:** Code of the transect where data were collected.
  ○ **Date:** Date of data collection.
  ○ **Weather:** Weather situation around the time of observation.
  ○ **Start_time:** Local time when data collectors and assistants started walking the transect.
  ○ **End_time:** Local time when data collectors and assistants finished walking the transect.
  ○ **Location_along transect (m):** Location along the 1 km transect where data were collected.
  ○ **Type_of_animal_sign:** Type of animal sign observed.
  ○ **Local_name:** Local name of the animal whose sign was observed.
  ○ **Perp_distance_from_transect(m):** Perpendicular distance between the transect and observed sign in metres.
  ○ **Vegetation_type:** Code of vegetation type where the sign was observed.
  ○ **Canopy_openness:** Estimation of the openness of the canopy classified as “open”, “average”, or “closed”.
  ○ **Understorey_openness:** Estimation of the openness of the understorey classified as “open”, “average”, or “closed”.
  ○ **Visibility(m):** Horizontal visibility in the understorey in metres.
‐ File: **Direct observations of great ape nests**
  ○ **Data Collector:** Person(s) in charge of data collection.
  ○ **Assistant:** Person(s) assisting with data collection.
  ○ **Study_site:** Site of data collection.
  ○ **Transect_number:** Number of the transect where data were collected.
  ○ **Transect:** Code of the transect where data were collected.
  ○ **Date:** Date of data collection.
  ○ **Weather:** Weather situation around time of observation.
  ○ **Start_time:** Local time data collectors and assistants started walking the transect.
  ○ **End_time:** Local time data collectors and assistants finished walking the transect.
  ○ **Breaks(min):** Number of minutes that were used to take breaks while walking the transect.
  ○ **Nest_site_code:** Unique code for each site where great ape nests were found. “GO” in the code indicates gorilla nests and “CH” indicates chimpanzee nests.
  ○ **Nest_age_category:** Classification (1–6, where 6 is the oldest) of the age of every nest.
  ○ **Nest_code:** Unique code for every nest at each site.
  ○ **V/NV:** Visibility of the nest from the transect. *V* = visible, NV = not visible.
  ○ **Location_along_transect(m):** Location along the 1 km transect where data were collected.
  ○ **L/R:** Location of the nest on the left (L) or right (R) side of the transect.
  ○ **Perp_distance_from_transect(m):** Perpendicular distance between the transect and observed nest in metres.
  ○ **Nest_diameter(cm):** Diameter of the nest is centimetres.
  ○ **Nest_type:** Type of nests. It was indicated whether the nest was made of herbs, a mix of plant species, or constructed on the side or top of a tree.
  ○ **Nest_heigth(m):** Heigt of the nest, if arboreal, in metres.
  ○ **Tree_height(m):** Height of the tree in metres if the nest was built in a tree.
  ○ **Circumference(m):** Circumference of the tree in which the nest was built (in metres).
  ○ **Fruits_on_tree:** It was indicated whether the tree had fruits (Yes, No, or NA).
  ○ **Vegetation_type:** Code of vegetation type where the nest was observed.
  ○ **Canopy_openness:** Estimation of the openness of the canopy classified as “open”, “average”, or “closed”.
  ○ **Understorey_openness:** Estimation of the openness of the understorey classified as “open”, “average”, or “closed”.
  ○ **Visibility(m):** Horizontal visibility in the understorey in metres.
  ○ **Latitude:** Coordinates of latitude.
  ○ **Longitude:** Coordinates of longitude.
‐ File: **Direct observations of mammal signs**
  ○ **Data Collector:** Person(s) in charge of data collection.
  ○ **Assistant:** Person(s) assisting with data collection.
  ○ **Study_site:** Site of data collection.
  ○ **Transect_number:** Number of the transect where data were collected.
  ○ **Transect:** Code of the transect where data were collected.
  ○ **Date:** Date of data collection.
  ○ **Weather:** Weather situation around the time of observation.
  ○ **Start_time:** Local time data collectors and assistants started walking the transect.
  ○ **End_time:** Local time data collectors and assistants finished walking the transect.
  ○ **Breaks(min):** Number of minutes that were used to take breaks while walking the transect.
  ○ **Location_along_transect(m):** Location along the 1 km transect where data were collected.
  ○ **Local_name:** Name of detected species in local language.
  ○ **#_individuals:** Number of detected individuals per observation.
  ○ **Distance_observation:** Number of metres between the data collector and the observed species.
  ○ **Angle_observation:** Angle between the transect and the observed species during the observation.
  ○ **Vegetation_type:** Code of vegetation type where the sign was observed.
  ○ **Canopy_openness:** Estimation of the openness of the canopy classified as “open”, “average”, or “closed”.
  ○ **Understorey_openness:** Estimation of the openness of the understorey classified as “open”, “average”, or “closed”.
  ○ **Visibility(m):** Horizontal visibility in the understorey in metres.
‐ File: **Bird observations with the point counts method**
  ○ **Data Collector:** Person(s) in charge of data collection.
  ○ **Assistant:** Person(s) assisting with data collection.
  ○ **Study_site:** Site of data collection.
  ○ **Transect_number:** Number of the transect where data were collected.
  ○ **Transect:** Code of the transect where data were collected.
  ○ **Date:** Date of data collection.
  ○ **Weather:** Weather situation around time of observation.
  ○ **Count_station (m):** Station along the transect (0 m, 500 m, or 1000 m), where the observation was made.
  ○ **Direction_observation:** Compass bearing (0, 90, 180, or 270) of the direction in which the observation was made.
  ○ **Time_observation:** Time when the observation was made.
  ○ **Vegetation_type:** Code of vegetation type where the sign was observed.
  ○ **Canopy_openness:** Estimation of the openness of the canopy classified as “open”, “average”, or “closed”.
  ○ **Understorey_openness:** Estimation of the openness of the understorey classified as “open”, “average”, or “closed”.
  ○ **Visibility(m):** Horizontal visibility in the understorey in metres (estimated).
  ○ **Local_name_seen:** If species was seen, name of species in local language.
  ○ **#_individuals_seen:** Number of individuals of the species seen.
  ○ **Local_name_heard:** If species was heard, name of species in local language.
  ○ **#_individuals_heard:** Number of individuals of the species heard.
‐ File: **Bird observations with the continuous counts method**
  ○ **Data Collector:** Person(s) in charge of data collection.
  ○ **Assistant:** Person(s) assisting with data collection.
  ○ **Study_site:** Site of data collection.
  ○ **Transect_number:** Number of the transect where data were collected.
  ○ **Transect:** Code of the transect where data were collected.
  ○ **Date:** Date of data collection.
  ○ **Weather:** Weather situation around the time of observation.
  ○ **Start_time:** Local time data collectors and assistants started walking the transect.
  ○ **End_time:** Local time data collectors and assistants finished walking the transect.
  ○ **Breaks(min):** Number of minutes that were used to take breaks while walking the transect.
  ○ **Location_along_transect(m):** Location along the 1 km transect where data were collected.
  ○ **Vegetation_type:** Code of vegetation type where the sign was observed.
  ○ **Canopy_openness:** Estimation of the openness of the canopy classified as “open”, “average”, or “closed”.
  ○ **Understorey_openness:** Estimation of the openness of the understorey classified as “open”, “average”, or “closed”.
  ○ **Visibility(m):** Horizontal visibility in the understorey in metres (estimated).
  ○ **Local_name_seen:** If species was seen, name of species in local language.
  ○ **#_individuals_seen:** Number of individuals of the species seen.
  ○ **Distance_observation_seen:** Number of metres between the data collector and the species seen (estimated).
  ○ **Angle_observation_seen:** Angle between the transect and the species detected during the observation.
  ○ **Local_name_heard:** If the species was heard, name of the species heard in local language.
‐ File: **GPS coordinates of sensors**
  ○ **Transect:** Transect where the sensor was deployed.
  ○ **X_Sensor:** Latitude of the location where the sensor was deployed.
  ○ **Y_Sensor:** Longitude of the location where the sensor was deployed.
  ○ **Remoteness (Distance to village [m]):** Distance between the sensor and the nearest village in metres.
  ○ **Accessibility (distance to trail [m]):** Distance between sensor and nearest trail in metres.
‐ File: **Temperature**
  ○ **Date:** Date of measurement.
  ○ **Time:** Time of measurement. Measurements were made every hour for 24 h a day. There is one column for each hour.
  ○ **MAX:** Maximum temperature for the day.
  ○ **MIN:** Minimum temperature for the day.
  ○ **AVERAGE:** Average temperature for the day.
‐ File: **Moon phases**
  ○ **Date:** Date of observation.
  ○ **Day:** Day of the month when the observation was made.
  ○ **Month:** Month when the observation was made.
  ○ **Year:** Year when the observation was made.
  ○ **Sky:** Amount of clouds in the sky during the observation.
  ○ **Moon_phase:** Moon phase during the observation.
‐ File: **Humidity**
  ○ **Date:** Date of measurement.
  ○ **Time:** Time of measurement. Measurements were made every hour for 24 h a day. There is one column for each hour.
  ○ **MAX:** Maximum humidity for the day.
  ○ **MIN:** Minimum humidity for the day.
  ○ **AVERAGE:** Average humidity for the day.
‐ File: **Rainfall**
  ○ **Date:** Date of measurement.
  ○ **Rainfall(mm):** Amount of precipitation measured in millimetres.

## 2 Experimental Design, Materials, and Methods

‐ Data were obtained in three study sites that differ in land-use type and conservation management in the northern part of the Dja Faunal Reserve's buffer zone in Cameroon.
‐ Data were collected between February and May 2020.
‐ In every study site, 6 transects of 1 km each were opened.
‐ Audio data were obtained using the following procedure:
  ○ An AudioMoth bioacoustics sensor was deployed in the middle of every transect.
  ○ The sensors were deployed at a height of 2 m at a 90° orientation.
  ○ The sensors were kept in zip lock bags within a protective case, with a small hole at the location of the sensors’ microphone, to protect them from rain and animals.
  ○ All sensors were set to record the first minute of every hour at 48 kHz and 30.6 dB.
  ○ Recordings made during the night were screened beforehand. Only night recordings with vocalisations from other species than easily recognisable insects, amphibians, or western tree hyraxes were played to the local experts.
  ○ Recordings were played to two local experts who identified all audible species.
  ○ Names of the vocalising species were noted down in Badjué (local language).
  ○ Vocalisations of birds and mammals were identified by species. Vocalisations of insects and amphibians were identified by class. Unidentifiable vocalisations were recorded as “Animal unknown” or “Bird unknown”.
‐ Field surveys were conducted to collect data on anthropogenic and ecological factors:
  ○ Vegetation types were described at every 50 m interval in each transect.
  ○ Human activity was described by identifying all human signs within a 2 m range perpendicular to each transect. For each sign, the location along the transect and the vegetation type were recorded.
  ○ Mammal activity was described indirectly by identifying animal signs within 2 m on either side of the transect and corresponding location along the transect, vegetation type, canopy openness, understorey openness and horizontal visibility. The local guide identified the type of animal sign and local name of the species. All transects were surveyed twice. Rainfall between the two surveys prevented overlap.
  ○ Presence of great apes, central chimpanzees (*Pan troglodytes*) and western lowland gorillas (*Gorilla gorilla*), was described by the observation of their nests along the transects. For every nest, age, location along the transect (m), perpendicular distance from the transect (m), and circumference (cm) were recorded. Furthermore, vegetation type, canopy openness, understorey openness, horizontal visibility (m), and coordinates were noted. Additionally, for gorillas, the type of nest was described by the composition of plants used for construction. For central chimpanzee nests, the type of nest was described by its position in the tree, an estimation of the height of the nest, an estimation of the height and circumference of the tree, and the presence or absence of fruits on the tree. These surveys were conducted twice for each transect, with one month in between surveys. No nests were counted twice.
  ○ Mammal activity was described directly by slowly walking along each transect (1 km/h). For all observed mammals, number, location along the transect (m), distance between the observer and the animal (m), angle of observation, vegetation type, canopy openness, understorey openness, and horizontal visibility (m) were recorded.
  ○ Bird activity was surveyed using point counts in fixed stations and direct observations. For the point counts, birds were recorded for 8 min at the start, middle, and end of each transect. An initial observation direction was randomly chosen and, after two minutes of observation, the observers rotated 90° in a clockwise direction. During direct observations, birds were recorded while walking the transect in the same manner as during the direct mammal surveys. For every observation, vegetation type, canopy openness, understorey openness, and horizontal visibility (m) were recorded.
  ○ Data on rainfall, humidity, and temperature were obtained in one site. Rainfall (mm) was measured daily. Temperature (°C) and humidity (RH) were measured hourly.
  ○ To assess additional anthropogenic factors, the shortest straight-line distance (m) between each sound recorder and the closest village and trail was measured using ArcGIS to get proxies for remoteness and accessibility, respectively.
‐ See Diepstraten et al. [2] for a detailed description of the experimental design for collecting these data.

## Ethics Statement

Our work did not involve the use of human subjects, animal experiments, or data collected from social media platforms.

## CRediT authorship contribution statement

**Johan Diepstraten:** Data curation, Formal analysis, Visualization, Writing – original draft, Writing – review & editing, Methodology, Conceptualization. **Jacques Keumo Kuenbou:** Supervision, Data curation, Methodology, Visualization. **Jacob Willie:** Conceptualization, Supervision, Methodology, Data curation, Formal analysis, Visualization, Writing – original draft, Writing – review & editing.

## Declaration of Competing Interest

The research was supported by the Antwerp Zoo Centre for Research and Conservation and the Association de la protection des grands singes. Stichting FONA and Stichting het Kronendak provided personal financial support to Johan Diepstraten. The authors declare that they have no known competing financial interests or personal relationships which have or could be perceived to have influenced the work reported in this article.
